# Beyond resection: imaging findings of expected and complicated postoperative changes in lung cancer

**DOI:** 10.1007/s11604-025-01818-1

**Published:** 2025-06-18

**Authors:** Makiko Murota, Takashi Norikane, Mariko Ishimura, Yuka Yamamoto, Riku Morita, Katsuya Mitamura, Yasukage Takami, Yuri Manabe, Mitsumasa Murao, Katashi Satoh, Naoya Yokota, Yoshihiro Nishiyama

**Affiliations:** 1 Department of Radiology, Faculty of Medicine, Kagawa University, 1750-1 Ikenobe, Miki-cho, Kita-gun, Kagawa 761-0793 Japan; 2 Department of Radiology, Diagnostic Imaging Center, Utazu Hospital, Utazu-cho, Ayauta-gun, Kagawa Japan; 3 Department of General Thoracic Surgery, Faculty of Medicine, Kagawa University, Kita-gun, Kagawa Japan

**Keywords:** Lung cancer, Chest CT, Thoracic surgery, Postoperative imaging, Surgical complications, Radiologic interpretation

## Abstract

Lung cancer remains the leading cause of cancer-related mortality, with surgical resection as the primary curative treatment for early-stage non-small cell lung cancer. However, distinguishing normal postoperative changes from complications on chest radiographs and CT scans presents a significant diagnostic challenge, necessitating precise radiologic interpretation. Postoperative complications manifest across a broad spectrum of timing and severity. Early complications include persistent air leak, pneumonia, and bronchopleural fistula, while late complications include bronchial anastomotic stricture, lung herniation, and unilateral pleuroparenchymal fibroelastosis. In addition, rare but clinically significant complications, such as lobar torsion, acute exacerbation of interstitial pneumonia, and pulmonary vein stump thrombosis, warrant careful consideration due to their potential for severe morbidity. Accurate identification of expected postoperative imaging findings and complications is essential to ensuring timely diagnosis and preventing unnecessary interventions. This review synthesizes current knowledge on surgical procedures, expected postoperative imaging findings, and key complications to refine radiologists’ diagnostic acumen and ultimately improve patient outcomes.

## Introduction

Lung cancer remains the leading cause of cancer-related mortality worldwide [1, 2], with surgical resection serving as the cornerstone of curative treatment for early-stage non-small cell lung cancer (NSCLC) [3–5]. However, distinguishing normal postoperative changes from complications on imaging can be challenging, particularly on chest radiographs (CXRs) and computed tomography (CT), necessitating precise radiologic assessment.

This review illustrates the spectrum of expected postoperative imaging findings across different surgical techniques and delineates the radiologic features of both common and rare complications. Recognizing uncommon complications is essential, as their imaging manifestations can be clinically significant. By improving recognition of these patterns, radiologists can enhance diagnostic accuracy, facilitate timely intervention, and reduce unnecessary procedures.

## Types of lung cancer surgery

### Standard surgical techniques for lung cancer (Table 1)

**Table 1:** Surgical procedures for lung cancer

Procedure	Description
Pneumonectomy	Reserved for cases where cancer involves proximal bronchi or vascular structures. Due to its association with high perioperative mortality and long-term postoperative complications, it is performed only when necessary
Lobectomy	Involves the resection of a single lobe along with its regional lymph nodes. It is widely considered the standard surgical approach for resectable lung cancer
Segmentectomy	A lung-preserving procedure guided by the segmental bronchi and vessels. In selected cases, sub-segmentectomy may be performed to further preserve pulmonary function
Wedge resection	A non-anatomic sub-lobar procedure that removes a small, peripheral portion of the lung parenchyma without regard to segmental boundaries

Surgical resection remains the definitive treatment for stages I, II, and selected IIIA NSCLC, often complemented by adjuvant systemic therapy offered in appropriate cases [3]. Lobectomy and regional lymph node dissection are the standard approaches, offering optimal oncologic outcomes. Emerging evidence suggests that in carefully selected stage I tumors (< 2 cm), anatomical segmentectomy provides locoregional disease control and overall survival comparable to those achieved with lobectomy [4, 5].

Sleeve lobectomy is indicated when malignancy extends to or near the bronchial bifurcation, most frequently involving the right upper lobe. Although pneumonectomy is generally avoided due to its elevated perioperative mortality (6–8%) and risk of long-term postoperative complications, it remains necessary for tumors invading proximal bronchi or vascular structures [6].

Minimally invasive techniques, including video-assisted thoracoscopic surgery (VATS) and robot-assisted thoracoscopic surgery (RATS), have gained prominence in recent years. [7]. VATS offers advantages such as reduced postoperative pain, preservation of lung function, and a lower incidence of postoperative complications [7]. RATS provides a three-dimensional surgical view and employs articulated instruments, allowing greater maneuverability within the thoracic cavity. These features may reduce postoperative complications and improve lymph node dissection [7].

## Expected postoperative imaging findings

### Imaging modalities for postoperative assessment

For routine early postoperative surveillance, CXR is generally sufficient owing to its accessibility, low radiation exposure, and cost-effectiveness. However, chest CT is warranted when complications are suspected, such as unexplained opacities or vascular complications [6].

### Normal postoperative changes by surgical procedure

#### Pneumonectomy

CXRs typically demonstrate an air- and fluid-filled postpneumonectomy space, with the trachea maintaining a midline position. Over time, a progressive mediastinal shift toward the resection site occurs due to air resorption within the pleural cavity [8].

#### Lobectomy

**Fig. 1:**
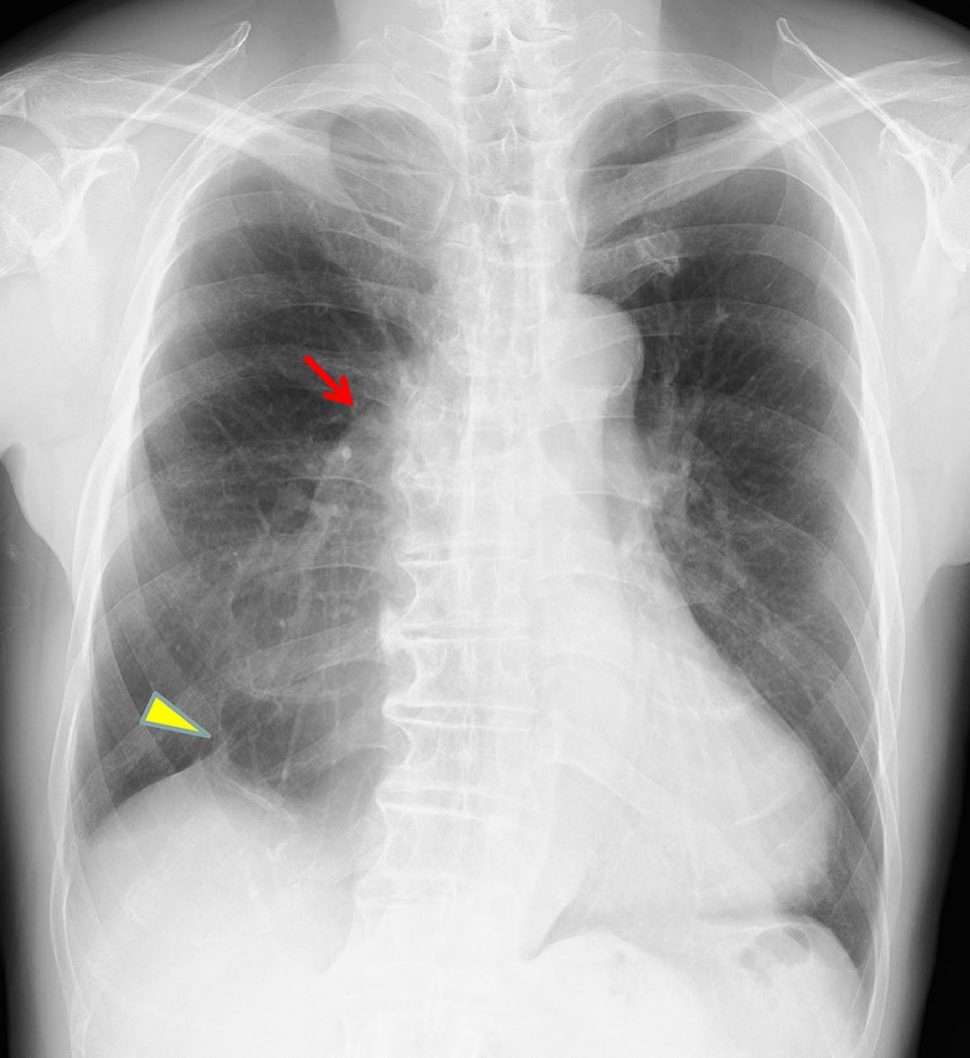
Normal postoperative changes on CXR following lobectomy. After right upper lobectomy, CXR demonstrates elevation of the right pulmonary hilum (arrow) and the presence of a juxtaphrenic peak sign (arrowhead). Recognizing these findings is essential, as they may mimic atelectasis

Following lobectomy, residual air in the pleural space resolves rapidly while pleural effusion gradually decreases. In some cases, a small, encapsulated effusion may persist long-term. Right middle lobectomy often results in minimal or absent volume loss due to the lung’s natural expansion dynamics [9]. Characteristic radiographic features include the juxtaphrenic peak sign and pseudo-lobar collapse, which may mimic atelectasis if the surgical history is unknown [6] (Fig. 1). The juxtaphrenic peak sign refers to a transient, triangular peak of the diaphragm, typically resulting from retraction of the remaining lower lobe and upward traction by the inferior accessory fissure or adjacent structures following upper lobectomy [10].

#### Segmentectomy

CT imaging reveals resection of the segmental bronchus, while the main lobar bronchus remains patent [9]. Similar to wedge resection, surgical staples are commonly visible along the resection margins on both CXR and CT.

#### Wedge resection

On postoperative imaging, wedge resection is characterized by linear areas of increased density corresponding to the surgical staple line, with or without adjacent soft-tissue thickening [6].

### Foreign body and post-surgical materials

#### Surgical materials and fat pad (Fig. 2)

**Fig. 2:**
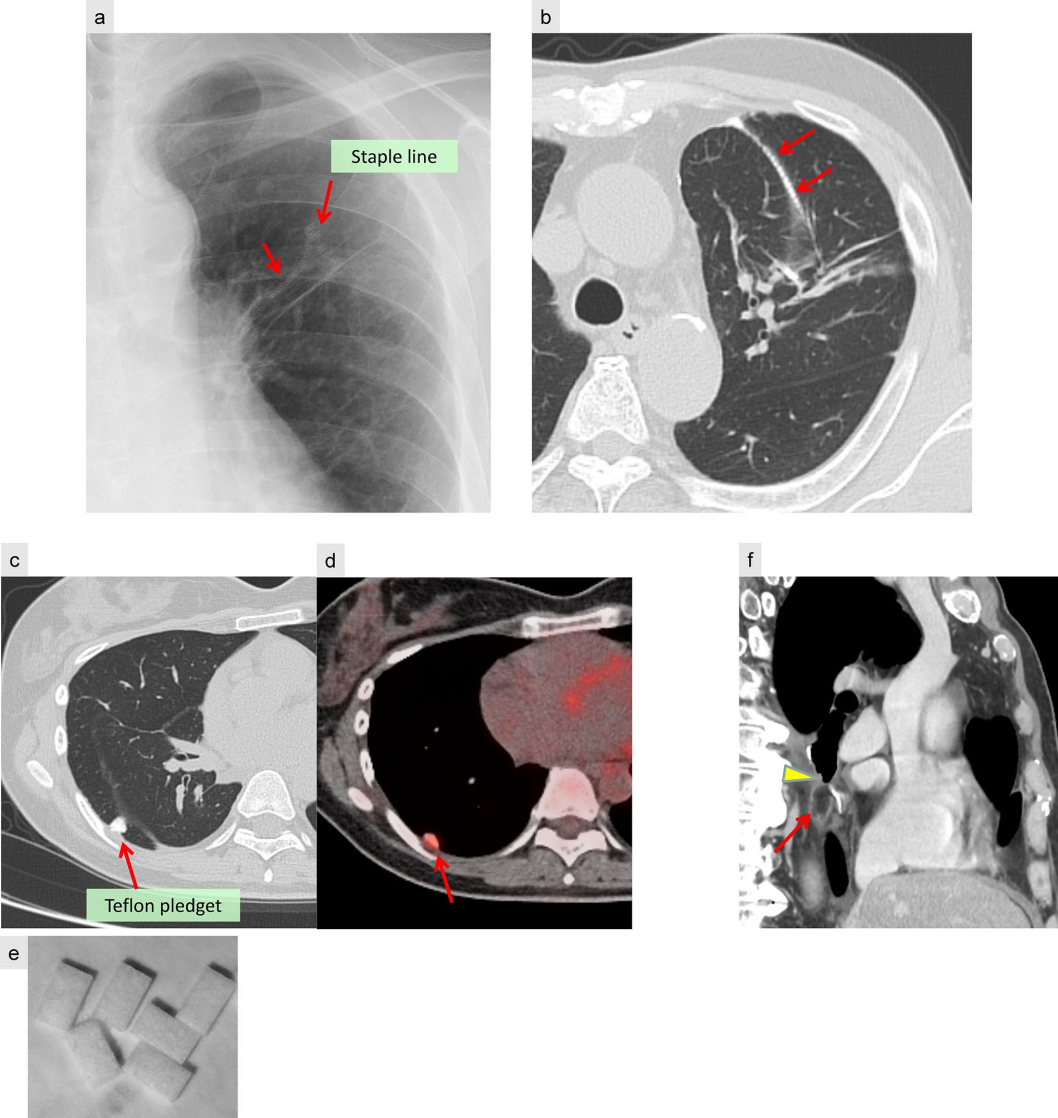
Surgical materials and fat pad. a, b After left S3 segmentectomy, CXR (a) and CT (b) reveal a linear high-attenuation structure corresponding to the staple line (arrow), indicative of suture materials. c, d, e Following right lower lobectomy, the Teflon pledget appears as a polygonal nodule with high attenuation on CT lung window settings (c), positioned in contact with the pleura and interlobar pleura. FDG-PET imaging demonstrates associated radiotracer accumulation (d). A photograph of the Teflon pledget is also provided (e). After right lower lobectomy, the bronchial stump is covered by free pericardial fat tissue (f). The sagittal CT image reveals a soft-tissue mass with fat attenuation (arrow) at the distal end of the bronchial stump (arrowhead)

Surgical materials are commonly used in lung resection to reinforce bronchial stumps and prevent postoperative complications. These include:

- Surgical staples, commonly used in segmentectomy, wedge resection, and lobectomy, appear as high-attenuation linear structures on imaging (Fig. 2a, b).
- Teflon pledgets are polygonal in shape and positioned adjacent to the pleura or bronchial stump to mitigate the risk of lobar torsion or bronchopleural fistula [11]. Various empirical techniques, such as inter-lobar fixation, chest well fixation, and the use of pledgets, have been employed to prevent lobar torsion [12, 13]. These can be misinterpreted as new nodular opacities or metastatic lesions, particularly on FDG-PET scans, which may demonstrate mild radiotracer uptake (Fig. 2c, d, e).
- Muscle flaps and pericardial fat pads, which are frequently employed to reinforce bronchial stumps, reduce the risk of postoperative airway leaks. On CT, pericardial fat appears as a soft-tissue mass, particularly after lobectomy or pneumonectomy (Fig. 2f).

Recognizing the postoperative imaging appearance of surgical materials, such as Teflon pledgets, muscle flaps, and pericardial fat pads, is essential to avoid misinterpretation as tumor recurrence or other complications. This recognition can help prevent unnecessary diagnostic procedures and interventions.

## Postoperative complications: imaging and clinical correlation

### Early postoperative complications (< 30 days post-surgery)

Complications following thoracic surgery can be broadly classified based on their timing: early complications, which manifest within the first 30 days postoperatively (e.g., persistent air leak, atelectasis, and pneumonia); late complications, which develop beyond this period (e.g., bronchial anastomotic stricture, lung herniation); and complications that may arise at any stage, such as bronchopleural fistula and empyema [14] (Table 2).

**Table 2:** Postoperative complications after lung cancer surgery

Early complications	Late complications	Complications occurring at any stage
Persistent air leak	Bronchial anastomotic stricture	Bronchopleural fistula
Atelectasis	Lung herniation	Empyema
Pneumonia	Chronic expanding hematoma	Chylothorax
Hemothorax	Local tumor recurrence	Thrombosis in the pulmonary vein stump
Lung torsion	Unilateral pleuroparenchymal fibroelastosis	
Acute respiratory distress syndrome		
Acute exacerbation of interstitial pneumonia		
Acute mediastinitis, deep sternal wound infection		

#### Persistent air leak

Persistent air leak occurs when air continues to escape from the lung parenchyma into the thoracic cavity beyond the expected timeframe for resolution. While some degree of air leakage is common following lobectomy, it typically resolves within 24–48 h as the remaining lung expands to occupy the pleural cavity. Air leaks persisting beyond 5–7 days are classified as persistent and may be associated with increased morbidity and prolonged hospitalization [8, 14].

**Fig. 3:**
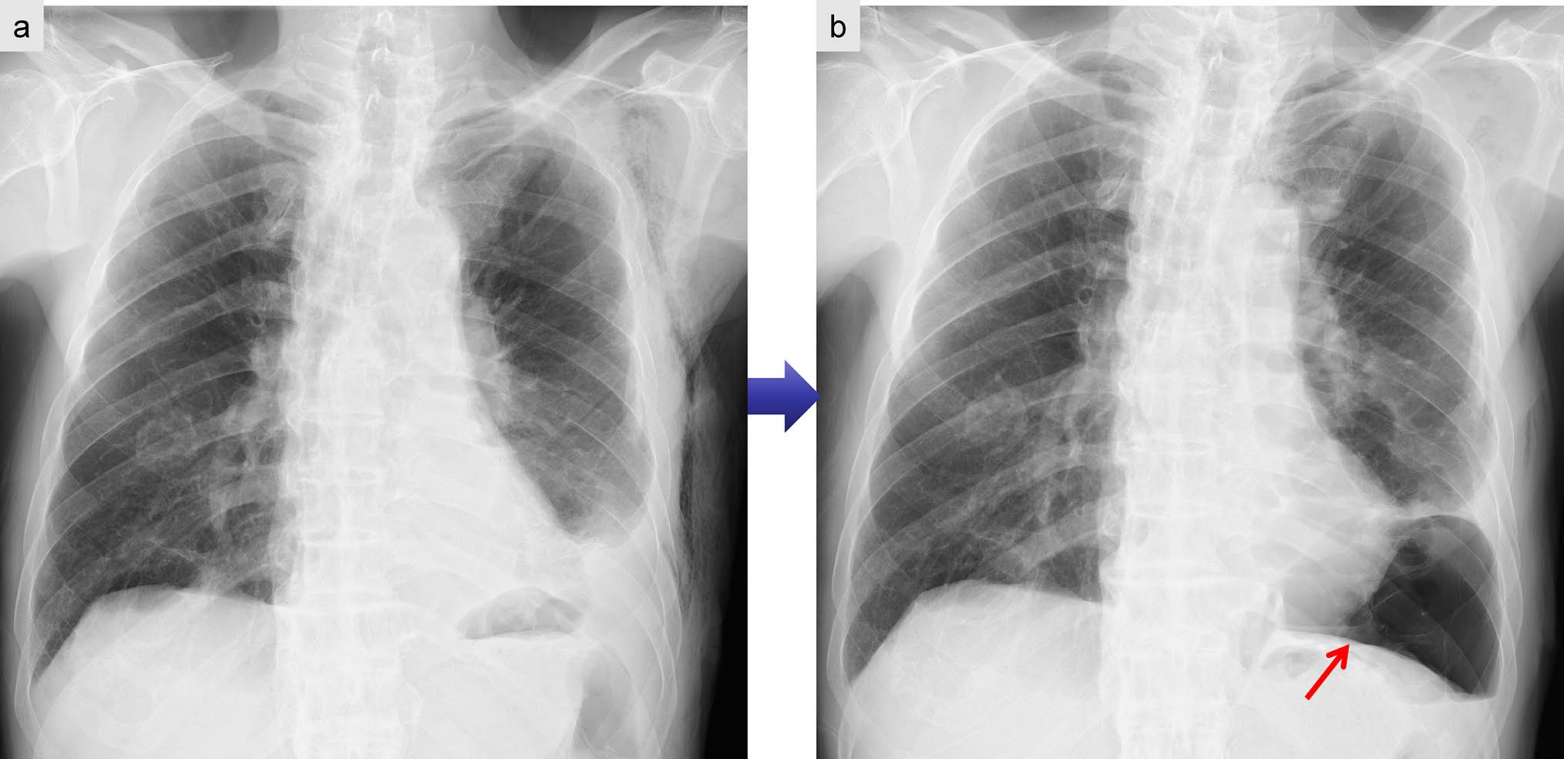
Persistent air leak following left lower lobectomy. CXR taken 1 week postoperatively appears unremarkable (**a**). However, a pneumothorax in the left lower lung field is evident 2 weeks after surgery (**b**)

CXRs and CT scans often reveal residual pneumothorax or subcutaneous emphysema [14, 15] (Fig. 3).

#### Atelectasis

**Fig. 4:**
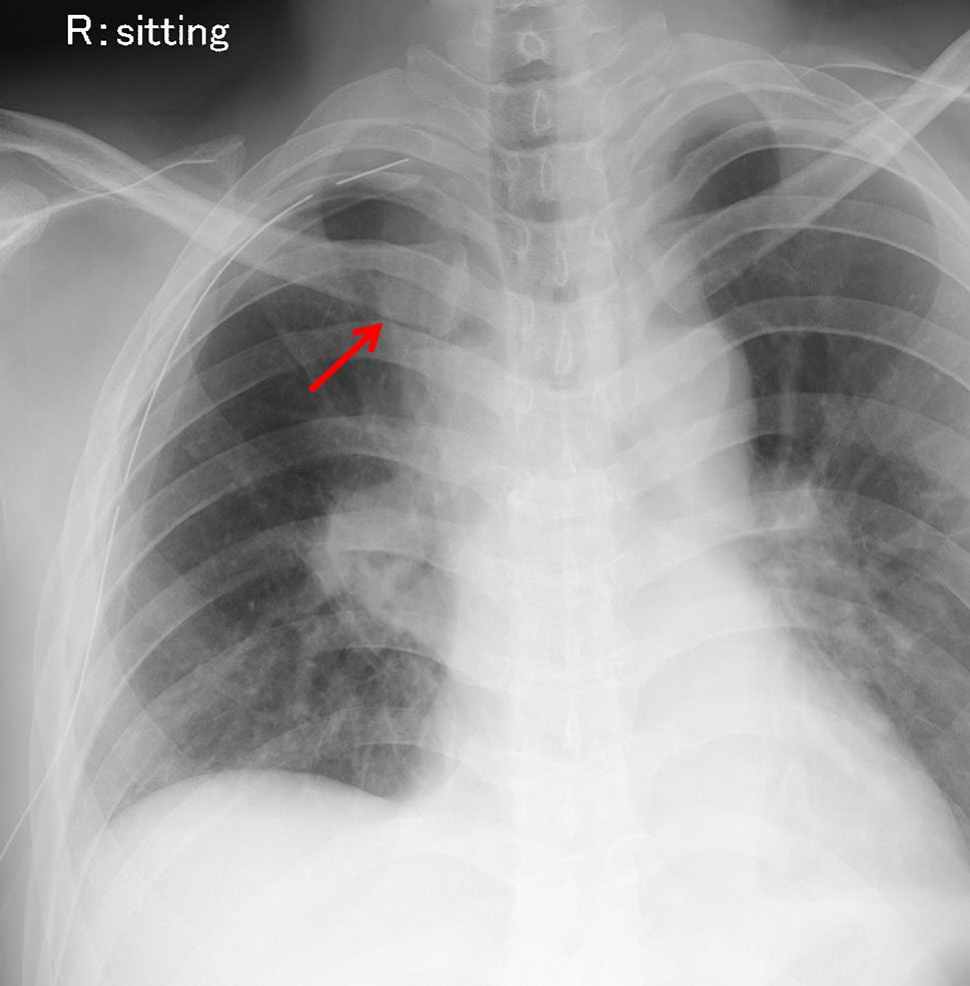
Atelectasis following right upper lobectomy. CXR reveals atelectasis of the right middle lobe, manifesting as opacification along the mediastinal aspect of the right upper lung field

Atelectasis is the most frequently encountered complication after thoracic surgery, typically arising during the early postoperative period [9]. It is most often caused by airway obstruction from retained secretions, leading to collapse of segments of the remaining lung. In some cases, superimposed infection may develop [9, 16]. Radiographic features vary depending on the location and extent of the lung involvement [8] (Fig. 4). On CT, atelectasis appears as increased parenchymal opacity with displacement of adjacent fissures and the hemidiaphragm [16].

#### Pneumonia

**Fig. 5:**
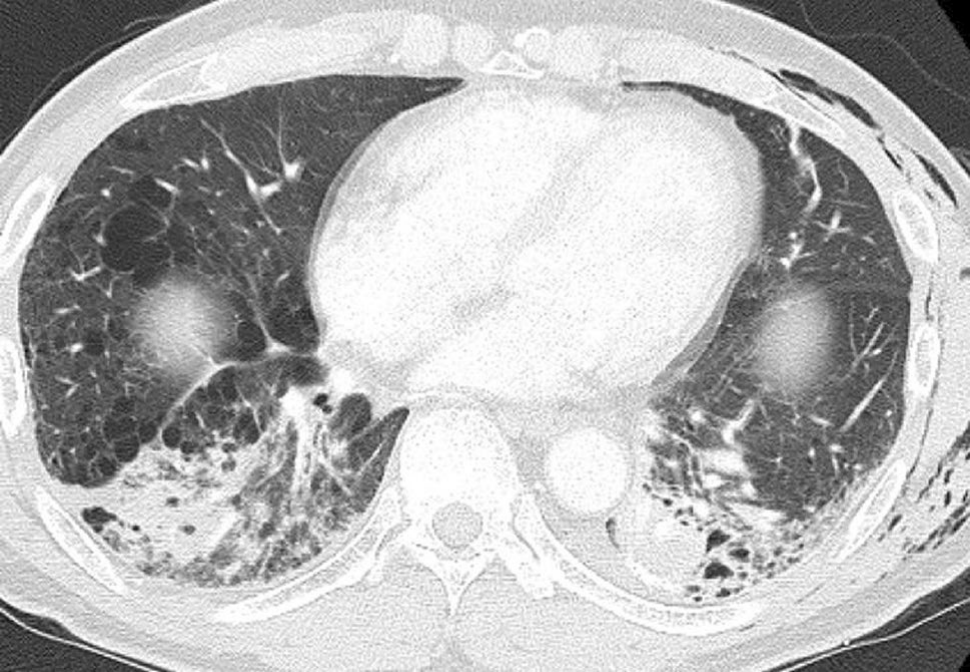
Pneumonia following left lower lobe wedge resection. The patient developed oxygen desaturation on postoperative day 1. Initial CXR was suggestive of pneumonia, and same-day CT imaging confirmed the diagnosis of aspiration pneumonia. CT revealed bilateral consolidation and ground-glass opacities predominantly in the lower lobes, consistent with aspiration pneumonia

Postoperative pneumonia is frequently associated with aspiration, mechanical ventilation, and inadequate pain control, all of which contribute to impaired pulmonary clearance and infection risk. On CXRs, pneumonia typically presents as patchy areas of consolidation, while CT provides better characterization, particularly in cases of aspiration pneumonia [9] (Fig. 5).

#### Hemothorax

Postoperative hemothorax arises from residual bleeding within the thoracic cavity, often due to persistent leakage from systemic thoracic vessels (e.g., bronchial and intercostal arteries), suture dehiscence of a pulmonary artery, or venous injuries. Hemorrhage from systemic arterial sources is the most frequent cause.

**Fig. 6:**
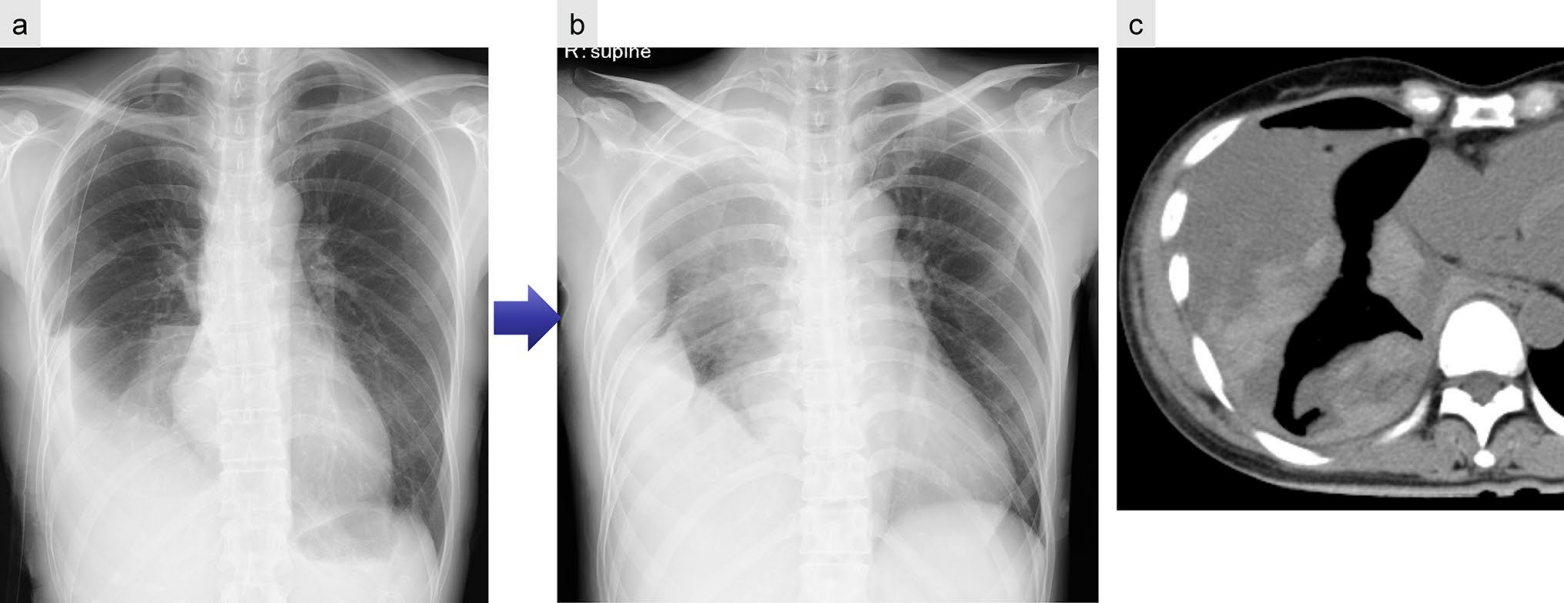
Hemothorax after right lower lobectomy. The patient developed hemorrhagic shock following the removal of the drainage tube. CXR after tube removal demonstrated a sudden decrease in tight-sided translucency (**a**, **b**). Urgent non-contrast CT revealed a pleural effusion containing high-attenuation areas, indicative of hemorrhagic fluid (**c**)

Radiographic findings on CXR include rapid opacification of the postoperative cavity. On non-contrast CT, hemothorax typically appears as a high-attenuation pleural effusion (~ 50 HU), often heterogeneous or with a fluid–fluid level [6] (Fig. 6). High attenuation within the effusion is strongly suggestive of a hematoma. However, findings may vary based on the timing of the bleed.

In cases where active bleeding is suspected, contrast-enhanced CT angiography can identify the source, and transcatheter embolization may be considered a therapeutic option.

#### Acute respiratory distress syndrome (ARDS)

Postoperative ARDS is most commonly observed following pneumonectomy and has historically been associated with poor prognosis [8]. However, recent studies indicate a reduced mortality rate of 40% to 45% [14].

**Fig. 7:**
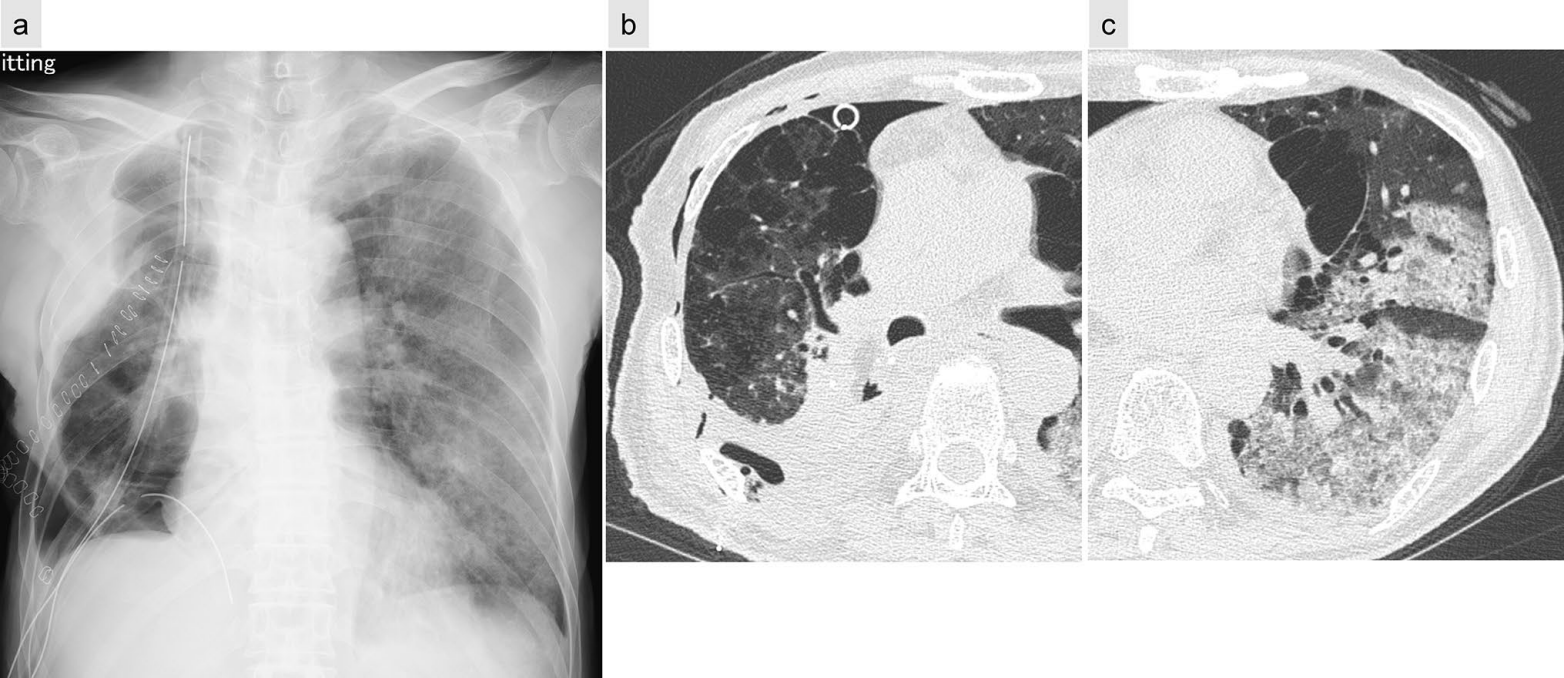
Acute respiratory distress syndrome (ARDS). Twenty-two days following right middle and lower lobe resection, follow-up CXR reveals diffuse ground-glass opacities in the left lung (**a**). CT imaging demonstrates extensive ground-glass opacities predominantly in the contralateral left lung rather than the postoperative right lung (**b**, **c**). Two days earlier, new opacities were noted in the left lung on CXR and CT (images not shown), prompting the initiation of antibiotic therapy. Despite oxygen administration at 8 L/min via face mask, the patient’s hypoxemia persisted, and the left lung opacities progressed. Given the clinical and imaging findings, a diagnosis of ARDS was established

Imaging findings include rapidly progressive bilateral opacities on CXR, while CT imaging reveals dorsal-predominant consolidation and ground-glass opacities (GGOs). These imaging features correspond to diffuse alveolar damage (DAD), the histopathologic hallmark of ARDS. During the organizing phases, traction bronchiectasis may also be present [8, 17, 18]. Unlike other causes of ARDS, postoperative ARDS may demonstrate asymmetric distribution, with relative sparing of the resected lung and predominant involvement of the contralateral lung [18] (Fig. 7). Radiologists play a critical role in identifying these characteristic imaging features, facilitating early detection of DAD and its distinction from other postoperative complications, such as infection or cardiogenic edema. Precise interpretation is vital for prompt management and improved clinical outcomes [8, 17].

#### Postoperative acute exacerbation (AE) of interstitial lung disease (ILD)

ILD, particularly idiopathic pulmonary fibrosis (IPF), is frequently associated with lung cancer. In patients with pre-existing ILD, surgical resection is often selected with curative intent and to reduce complication risk despite available alternatives such as chemotherapy and radiotherapy. However, postoperative acute exacerbation (AE) of ILD is a well-documented complication.

With an incidence of 9.3% and a mortality rate of 43.9%, AE of ILD represents the leading cause of lung cancer surgery-related mortality [19]. The risk of AE increases with the extent of lung resection (bi-lobectomy/pneumonectomy > segmentectomy/lobectomy > wedge resection) [19, 20].

**Fig. 8:**
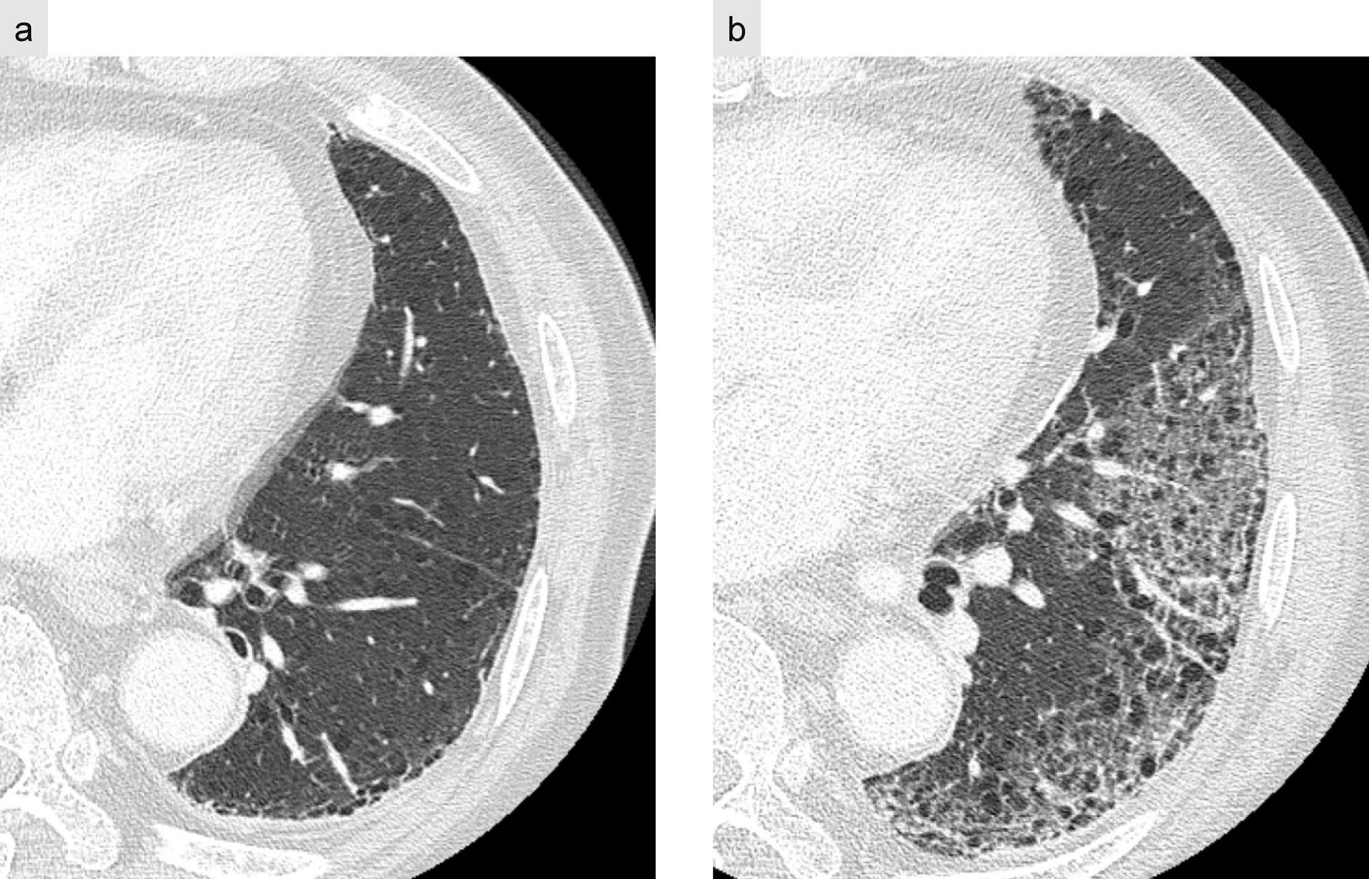
Postoperative acute exacerbation of idiopathic pulmonary fibrosis. Preoperative CT showed interstitial lung disease with the usual interstitial pneumonia pattern, including honeycombing (**a**). Four days after right upper lobectomy, the patient developed significant hypoxemia, prompting CT imaging. High-resolution CT reveals newly developed diffuse ground-glass and reticular opacities (**b**)

Imaging predictors for AE, such as preoperative CT findings of GGOs, consolidation, and pulmonary trunk enlargement, have been associated with an increased risk of AE [20]. The role of honeycombing on CT in predicting AE remains controversial [19–21]. Postoperatively, AE manifests as new GGOs and reticular opacities superimposed on pre-existing fibrosis (Fig. 8), resembling AE of ILD findings. Traction bronchiectasis and other contraction-related changes may also be present [22].

### Late postoperative complications (> 30 days post-surgery)

#### Bronchial anastomotic stricture

Bronchial anastomotic stricture is a late complication and the most frequently encountered complication following sleeve lobectomy, with an incidence of 18% [23]. The degree of stenosis and the presence of airway secretions determine whether distal lung collapse occurs.

**Fig. 9:**
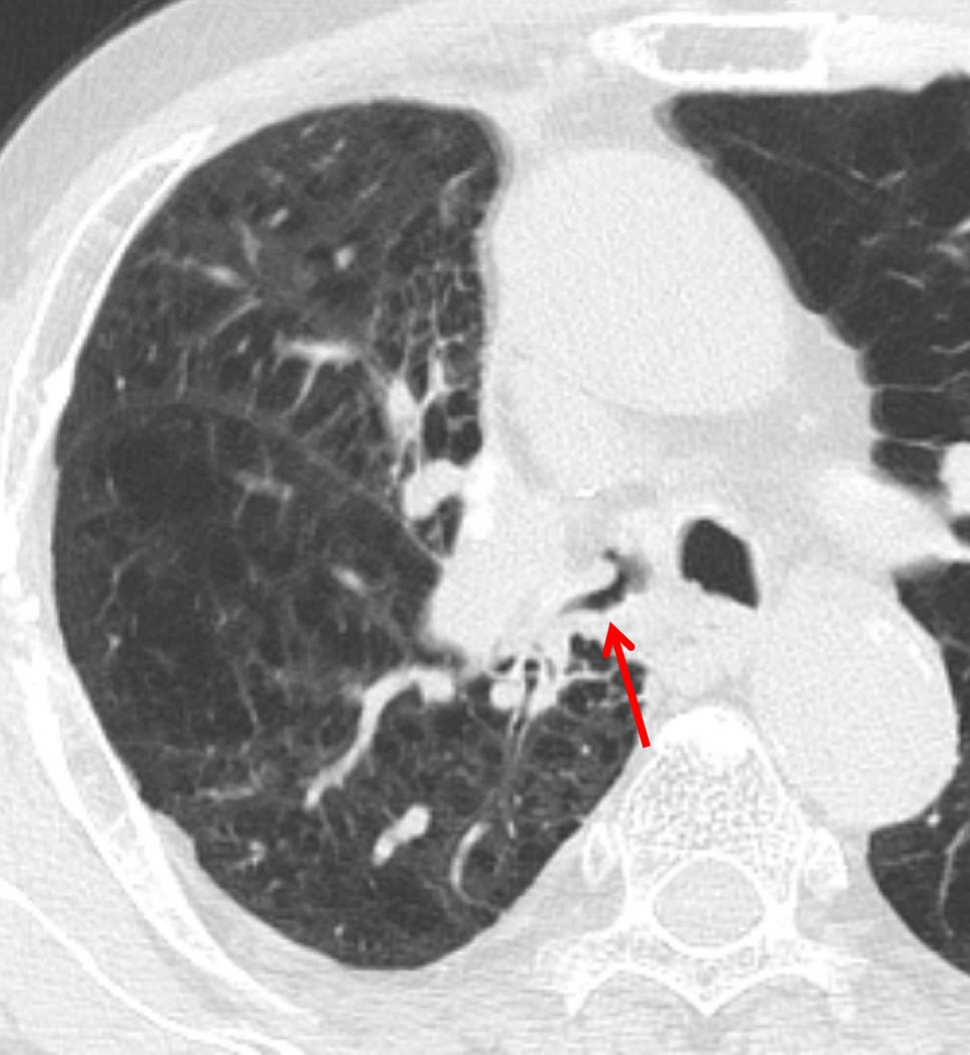
Bronchial anastomotic stricture. Four months after a right upper sleeve lobectomy for lung cancer, CT in the lung window demonstrates marked narrowing at the bronchial anastomotic site

Thin-section CT and multi-planar reconstructions are the most effective imaging modalities for evaluating airway narrowing and luminal obstruction [8] (Fig. 9).

#### Lung herniation

**Fig. 10:**
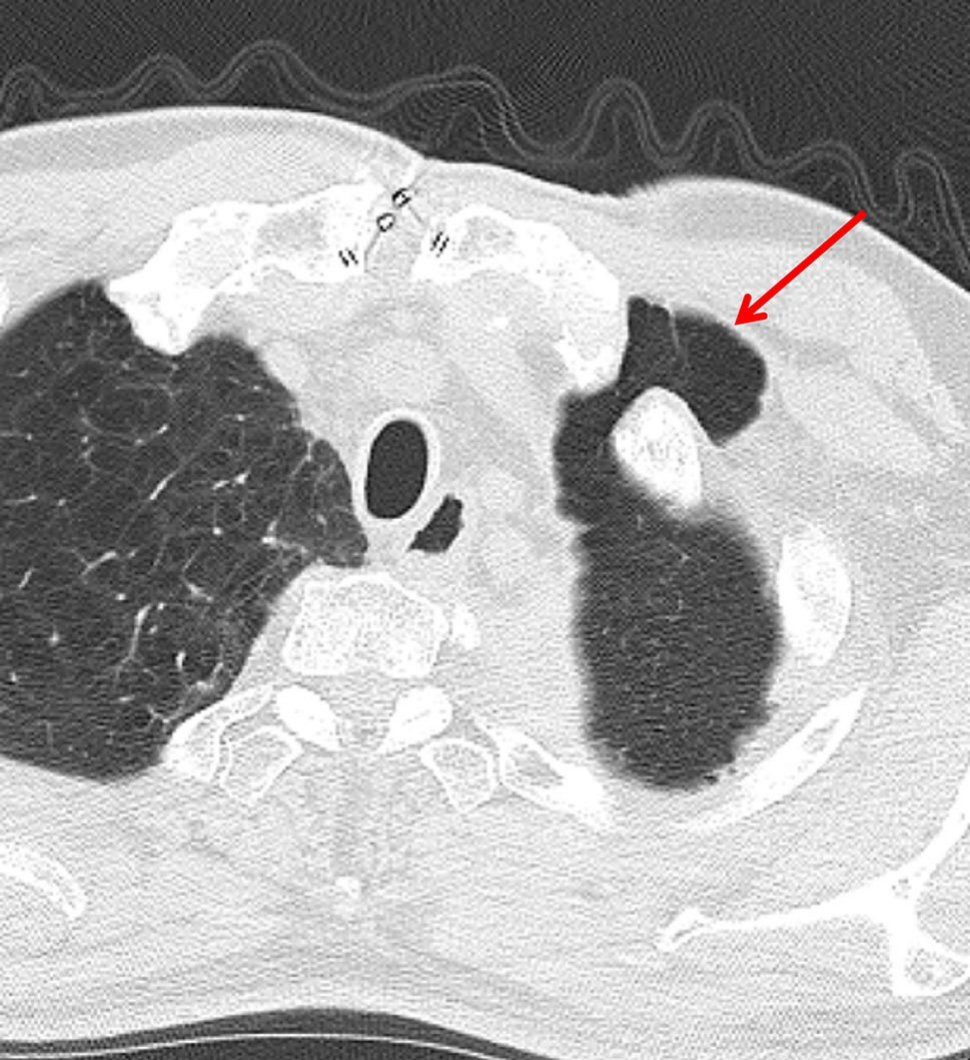
Postoperative lung hernia. Two years after left upper partial lobectomy, CT demonstrates lung parenchyma protruding through a chest wall defect at the left lung apex

Postoperative pulmonary hernia is a rare late complication in which the lung protrudes through a weakened region of the chest wall. While often asymptomatic, some patients present with a palpable or visible chest wall mass, which may become more prominent during coughing [24]. CT demonstrates lung parenchyma extending beyond the thoracic cavity, confirming the presence of the herniation (Fig. 10).

#### Chronic expanding hematoma

Chronic expanding hematomas are uncommon but clinically significant late postoperative complications. They may present as slowly enlarging, space-occupying lesions that persist over months to years and can mimic malignancy on imaging. These lesions are frequently associated with a history of thoracic surgery for conditions such as tuberculosis, pneumothorax, trauma, or tuberculous pleurisy [25].

**Fig. 11:**
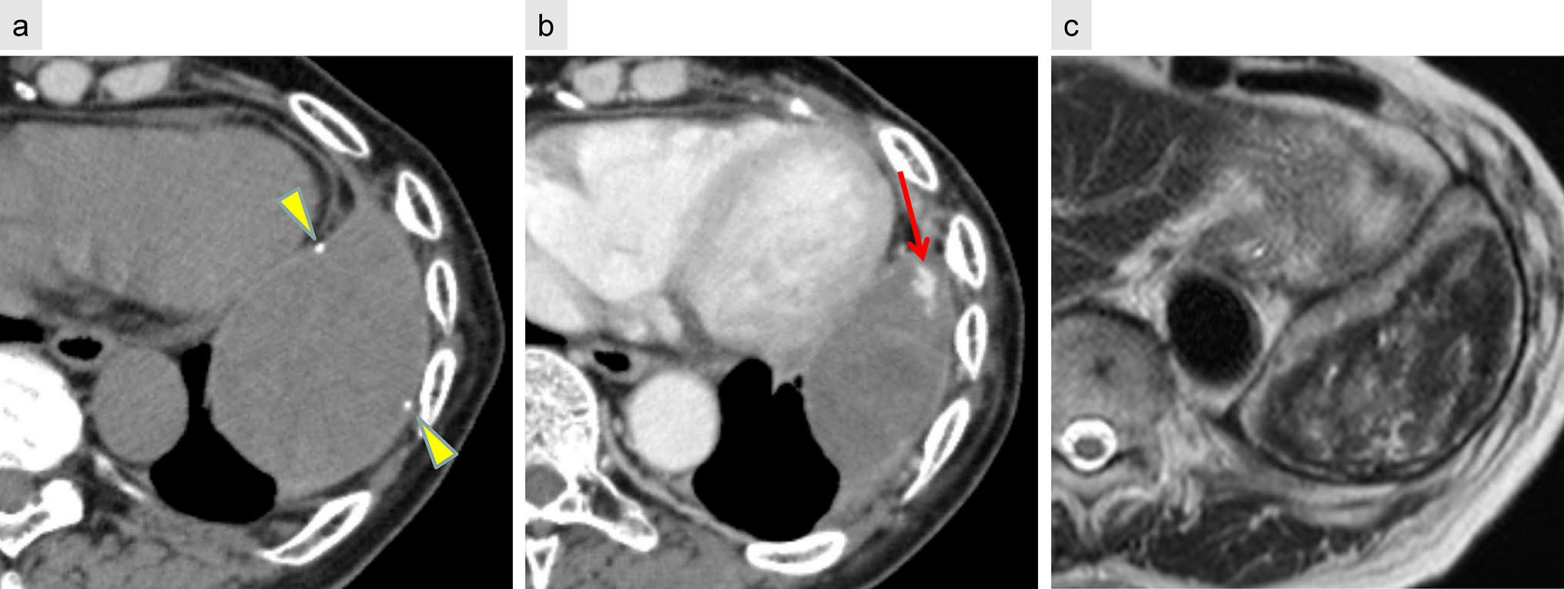
Chronic expanding hematoma. Five years following left lower lobectomy, non-contrast CT (**a**) reveals a mass with a focal calcified component (arrowhead) within the capsule. Contrast-enhanced CT (**b**) shows a small nodular enhancement at the lesion margin (arrow). T2-weighted MRI (**c**) demonstrates a mosaic pattern with areas of high and low signal intensity, characteristic of the “mosaic sign”

CT typically reveals a well-defined, encapsulated mass with peripheral calcification. Contrast-enhanced CT may demonstrate scattered small nodular enhancement at the lesion margins. On T2-weighted MRI, the “mosaic sign” has been reported as a characteristic feature, reflecting heterogeneous signal intensities due to the presence of both acute and chronic hemorrhagic components [26] (Fig. 11).

#### Local tumor recurrence

Postoperative local recurrence may involve bronchial stump recurrence, staple line recurrence within the lung parenchyma, mediastinal lymph node metastasis, pleural dissemination, and seeding along the surgical tract. These recurrences are most frequently observed within 2 years postoperatively [6]. Limited resections, such as segmentectomy or wedge resection, are associated with a higher recurrence rate than lobectomy.

**Fig. 12:**
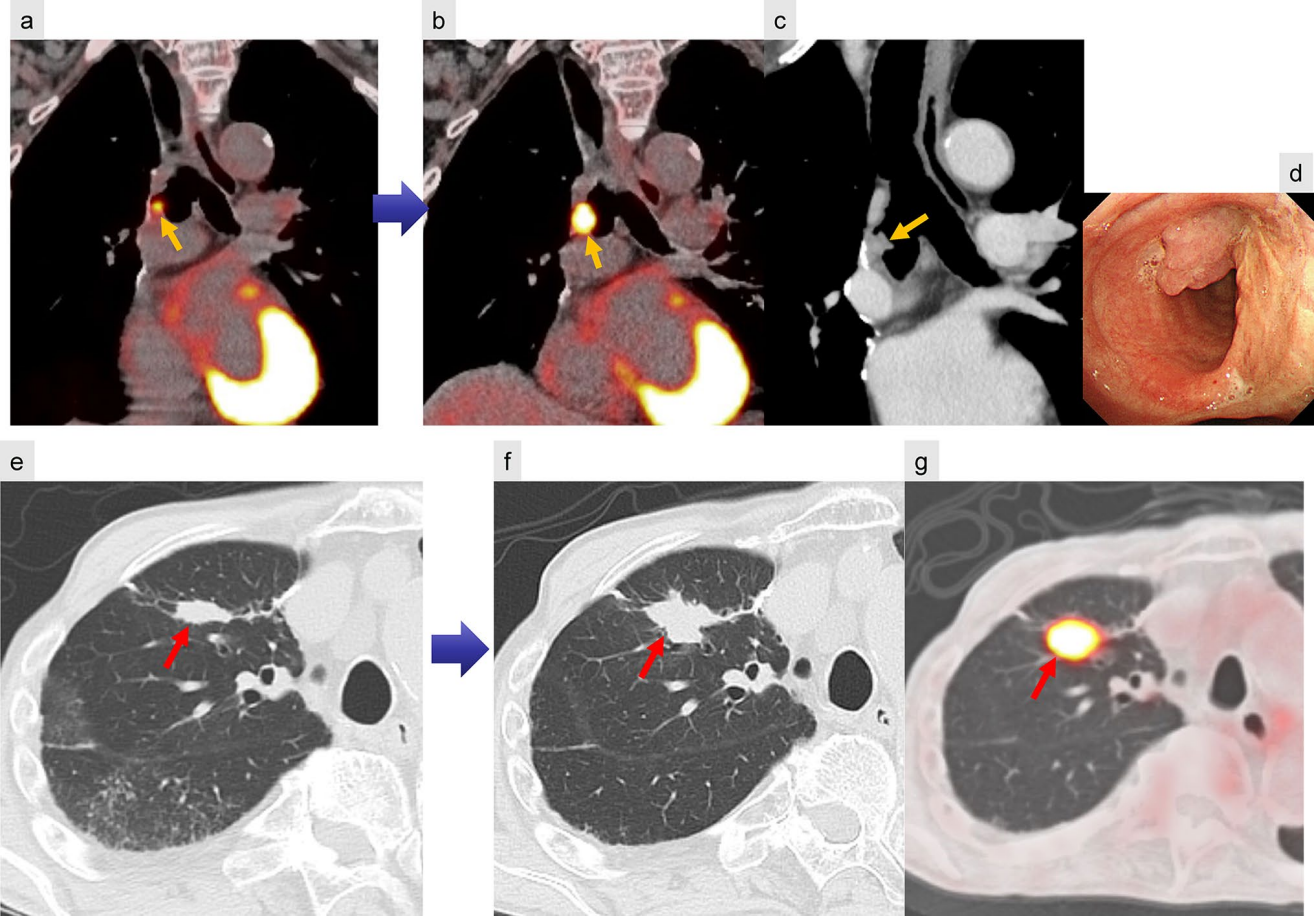
Local tumor recurrence. Bronchial stump recurrence after right upper and middle lobectomy (**a**–**d**). FDG-PET/CT performed 7 months postoperatively demonstrates increased FDG uptake in a small nodular lesion at the bronchial stump (**a**), which shows further progression at 13 months (**b**). CT reveals a soft-tissue mass protruding into the right bronchial lumen (**c**), and bronchoscopy confirms local recurrence (**d**). Staple line recurrence after partial resection of the right upper lobe lung cancer (**e**–**g**). Serial CT scans at 3 years (**e**) and 4 years (**f**) postoperatively demonstrate progressive soft-tissue enlargement along the staple line. FDG-PET/CT at 4 years shows abnormal FDG uptake consistent with tumor recurrence (**g**)

On CT, parenchymal recurrence near the resection margin is more frequently encountered after limited resection, such as segmentectomy or wedge resection, compared to lobectomy. Soft tissue growth on follow-up CT suggests recurrence at the bronchial stump, while staple line recurrence appears as focal soft-tissue enlargement along the resection margin. FDG-PET remains a valuable tool for detecting recurrent disease, with a reported accuracy of 94% (Fig. 12) [27].

**Fig. 13:**
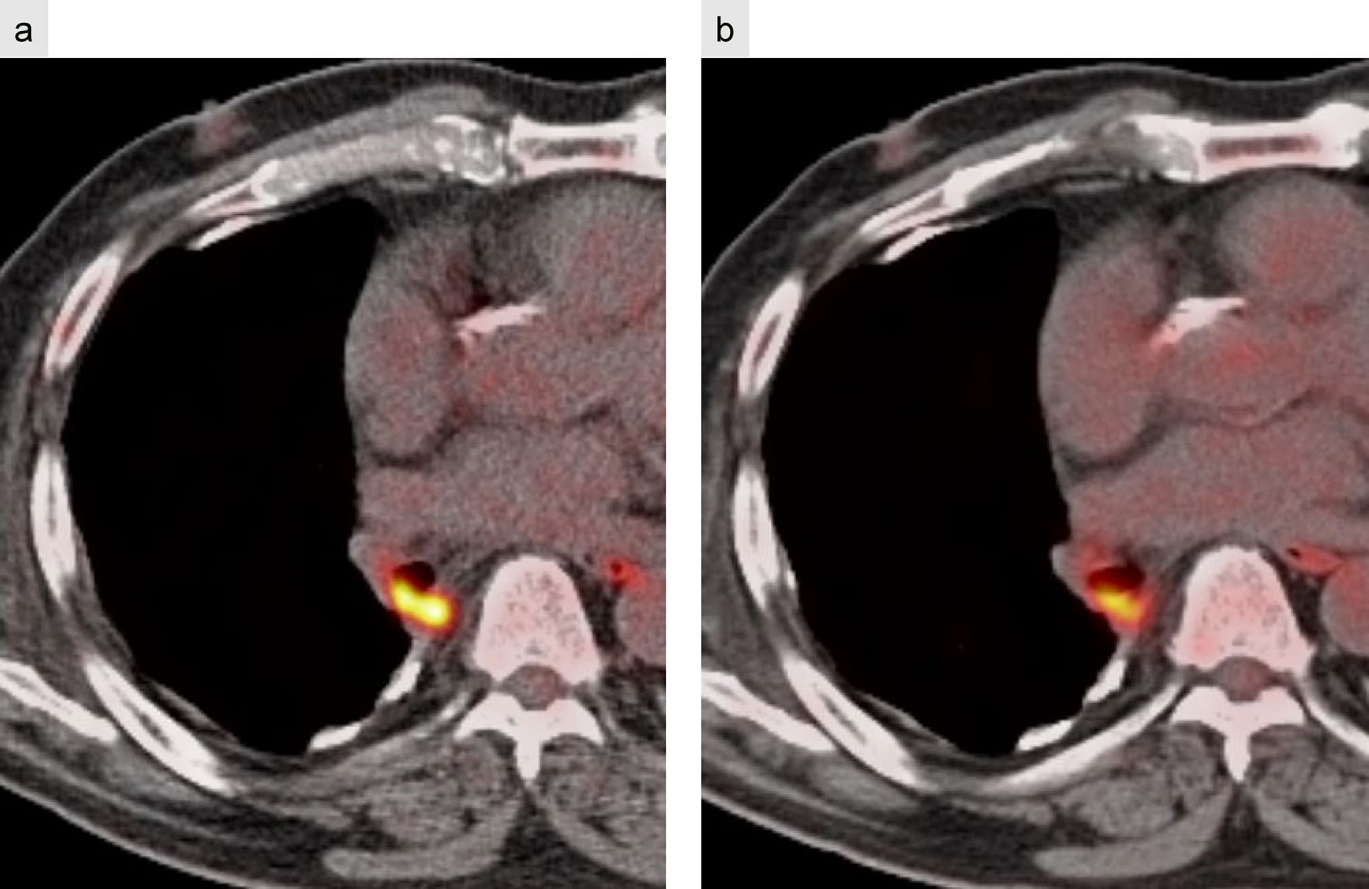
Staple line granuloma. FDG-PET/CT performed 3 years postoperatively shows increased FDG uptake at the bronchial stump (**a**). Bronchoscopy confirms no evidence of tumor recurrence. At the 5-year follow-up (**b**), FDG uptake persists but is slightly reduced, consistent with granulation tissue

However, postoperative inflammatory changes may also exhibit FDG uptake, complicating differentiation from malignancy. While inflammatory uptake typically subsides within 3 months and is rarely seen beyond 6 months, prolonged or worsening FDG accumulation has been documented, making definitive diagnosis challenging [28–30] (Fig. 13).

#### Unilateral pleuroparenchymal fibroelastosis (PPFE)

Unilateral PPFE is an infrequent but emerging and clinically relevant late complication following lung cancer surgery [31, 32], characterized by progressive fibrosis predominantly affecting the operated upper lung lobe. The incidence is approximately 4.3%, with a 10-year cumulative incidence of 5.3% [33]. Several risk factors have been identified, including male sex, lobar resection, the presence of a preoperative pulmonary apical cap, and reduced vital capacity (VC < 80%) [33].

Postoperative pleural effusion at 6 months has been recognized as a key early finding, often preceding the development of fibrotic changes [33]. As PPFE progresses, patients may experience worsening respiratory function and become more susceptible to chronic infections, such as pulmonary aspergillosis and nontuberculous mycobacterial infection. In some cases, this complication can contribute to significant morbidity and mortality, making early recognition crucial for distinguishing it from disease recurrence or other postoperative complications [33].

**Fig. 14:**
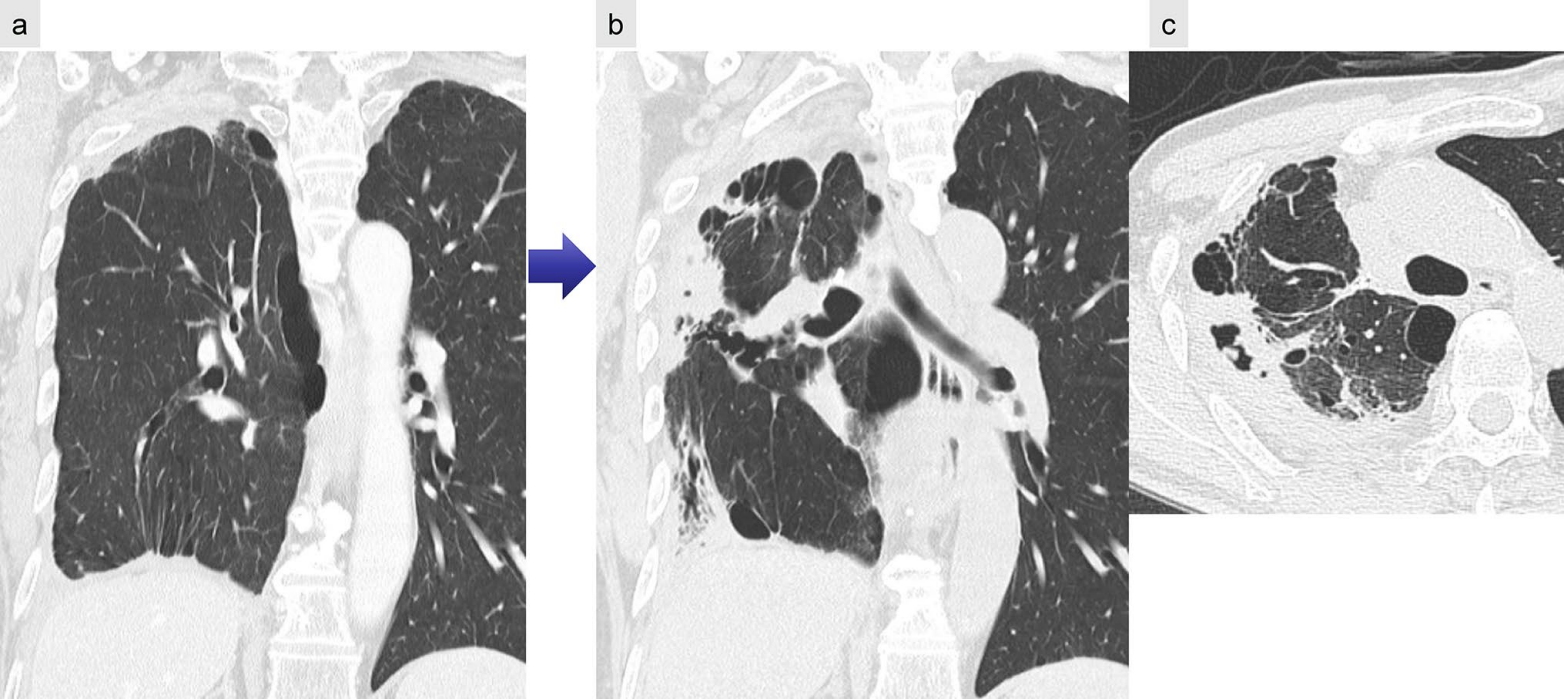
Unilateral pleuroparenchymal fibroelastosis. Seven years after right upper lobectomy, coronal CT images at postoperative year 1 (**a**) and year 7 (**b**) show progressive thickening of the apical cap, cystic changes in the right upper and middle lung fields, volume loss, and thoracic cage contraction. Axial CT at year 7 (**c**) reveals a nodule suspected to be a fungal ball within a sub-pleural cyst, leading to a diagnosis of aspergillosis

On CT, PPFE manifests as pleural thickening with adjacent sub-pleural fibrosis, predominantly affecting the operated upper lung field. As the disease advances, progressive volume loss, cystic changes, and thoracic deformity may develop, further compromising lung function (Fig. 14). The presence of a preoperative pulmonary apical cap and a persistent postoperative pleural effusion has been proposed as a potential predictor of this condition [33].

### Complications occurring at any stage

#### Bronchopleural fistula (BPF)

A BPF represents an abnormal communication between the bronchial tree and pleural space, occurring in early and late postoperative periods. BPFs are more frequently encountered following right pneumonectomy than left pneumonectomy, likely due to anatomical features of the right main bronchus—specifically, its shorter length, larger diameter, and reduced mediastinal coverage [6, 14]. These anatomic factors may also explain the increased use of bronchial stump reinforcement with fat tissue on the right side as a preventive measure against BPF formation.

**Fig. 15:**
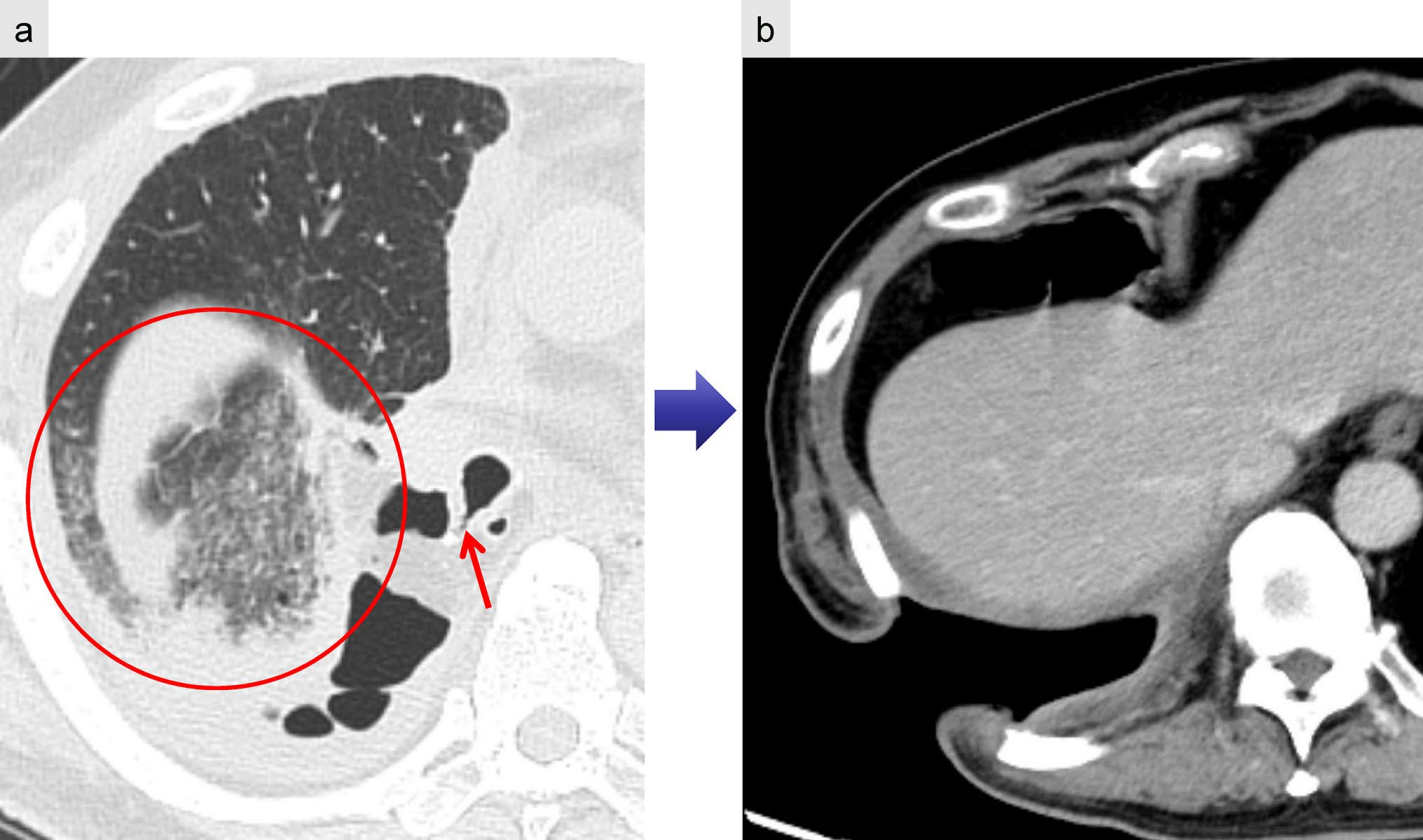
Bronchopleural fistula (BPF). Following right lower lobectomy (**a**, **b**), BPF with aspiration pneumonia and empyema are observed. CT (**a**) demonstrates a bronchial stump fistula (arrow) with adjacent air and fluid accumulation within the pleural cavity. The lung parenchyma shows ground-glass opacities (circle), consistent with aspiration pneumonia. An open-window thoracostomy was performed to manage the empyema (**b**)

CXR findings on imaging include decreased pleural fluid, persistent pneumothorax, or subcutaneous emphysema. CT is particularly valuable in identifying air-fluid levels within the pleural cavity, and in approximately 50% of cases, direct visualization of communication between the bronchus and pleural space can be identified [6] (Fig. 15a).

When empyema develops secondary to BPF, prompt management is critical. Initial treatment typically involves chest tube drainage and antimicrobial therapy to control infection and prevent contralateral contamination. In persistent or complex cases, advanced interventions such as open-window thoracostomy (Fig. 15b) or closure of the residual space with a muscle flap may be required [34]. These procedures aim to eradicate infection and eliminate the pleural dead space to prevent recurrence.

#### Empyema

Postpneumonectomy empyema is a severe complication and is associated with high mortality, with BPF as its primary etiology [15]. Early postoperative empyema is often due to residual pleural infection or intraoperative contamination, while late cases may develop months after surgery [9, 14].

**Fig. 16:**
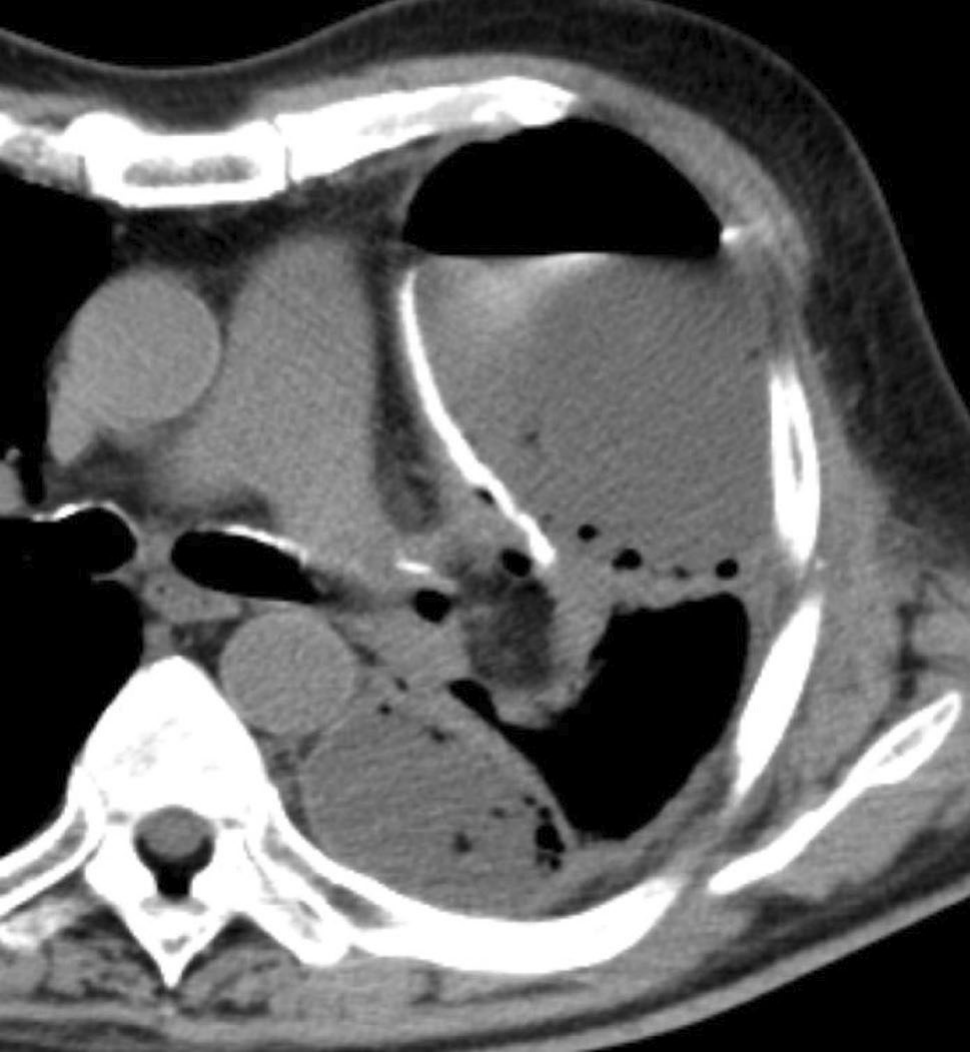
Empyema following left upper and lower segmentectomy to bronchopleural fistula. CT reveals fluid accumulation in the left pleural cavity, accompanied by adhesions and intrapleural air, resulting in a convex pleural contour

Radiologic findings include rapid pleural fluid accumulation, sometimes accompanied by a mediastinal shift. Fluid collections may exhibit convex protrusions with septations (Fig. 16). In cases where BPF coexists with empyema, pleural effusion near the bronchial stump may demonstrate air-fluid levels or small air bubbles [8, 9].

In patients with aspiration pneumonia, centrilobular opacities in the residual lung fields can serve as a diagnostic clue, further increasing the likelihood of empyema secondary to BPF (Fig. 15a).

#### Chylothorax

Chylothorax results from thoracic duct injury, leading to the accumulation of lymphatic fluid enriched with chylomicrons and lipids within the pleural space. The thoracic duct originates at the cisterna chyli, traverses the right aortic hiatus, and crosses leftward near the fifth thoracic vertebra before ascending into the mediastinum. Given this anatomical course, right-sided chylothorax is more commonly observed [14].

**Fig. 17:**
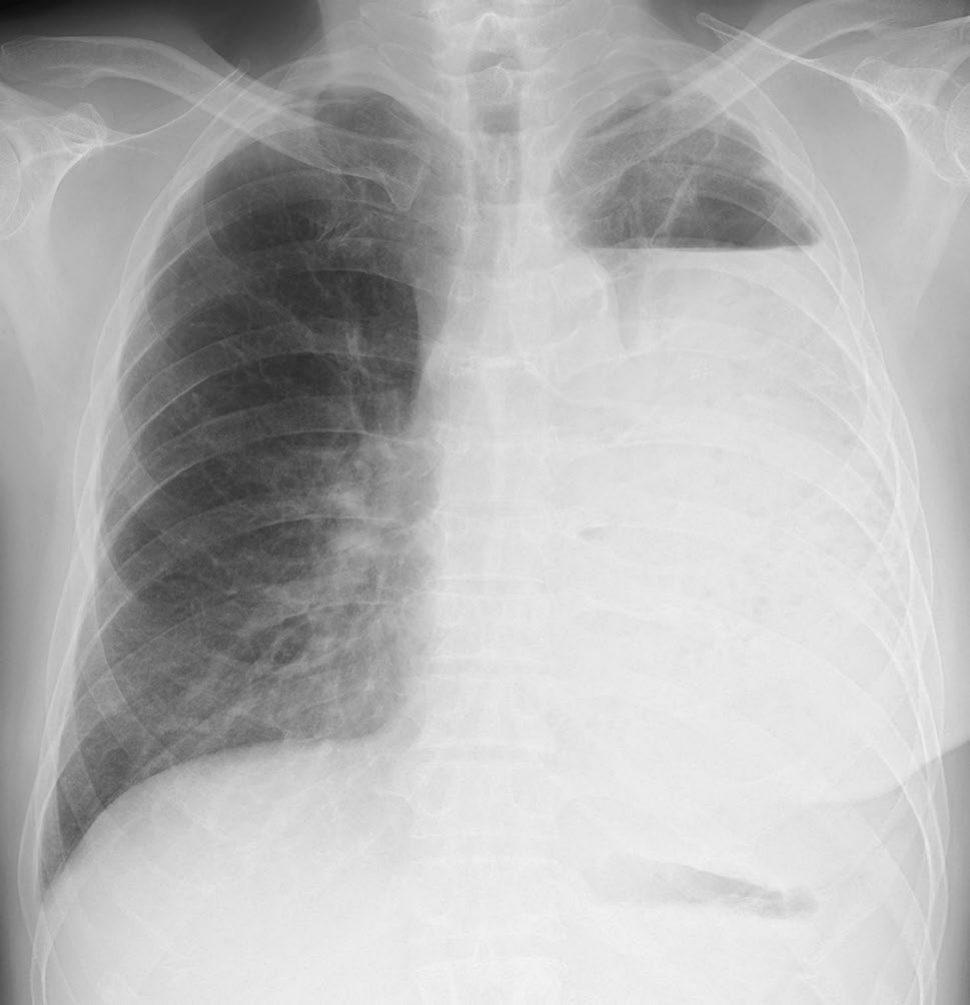
Chylothorax following left upper and lower lobe segmentectomy. CXR performed 2 weeks postoperatively demonstrates a large left pleural effusion. Thoracentesis confirmed chylothorax

CXR findings are often non-specific, demonstrating a progressively and rapidly enlarging pleural effusion (Fig. 17). On CT, the attenuation of the chylous effusion varies depending on composition: low attenuation is seen when lipid content is high, and higher attenuation may occur with elevated protein concentration.

However, CT alone often fails to differentiate chylous effusion from other causes of pleural effusion [8].

Management strategies range from conservative approaches (drainage) to interventional procedures (pleurodesis, surgical repair). Lymphangiography with embolization is a technique that allows both the identification of the leakage site and targeted treatment [8, 14, 15].

### Rare but critical complications

#### Lung torsion

Lung torsion is an extremely rare but life-threatening postoperative complication, occurring in less than 0.4% of cases. It is most commonly observed following right upper lobectomy (affecting the middle lobe) and upper division segmentectomy (affecting the lingula), occurring as an early complication postoperatively.

Torsion results in airway obstruction, disruption of the pulmonary arterial supply, and impaired venous drainage, which may lead to hemorrhagic infarction or parenchymal necrosis [8, 35].

**Fig. 18:**
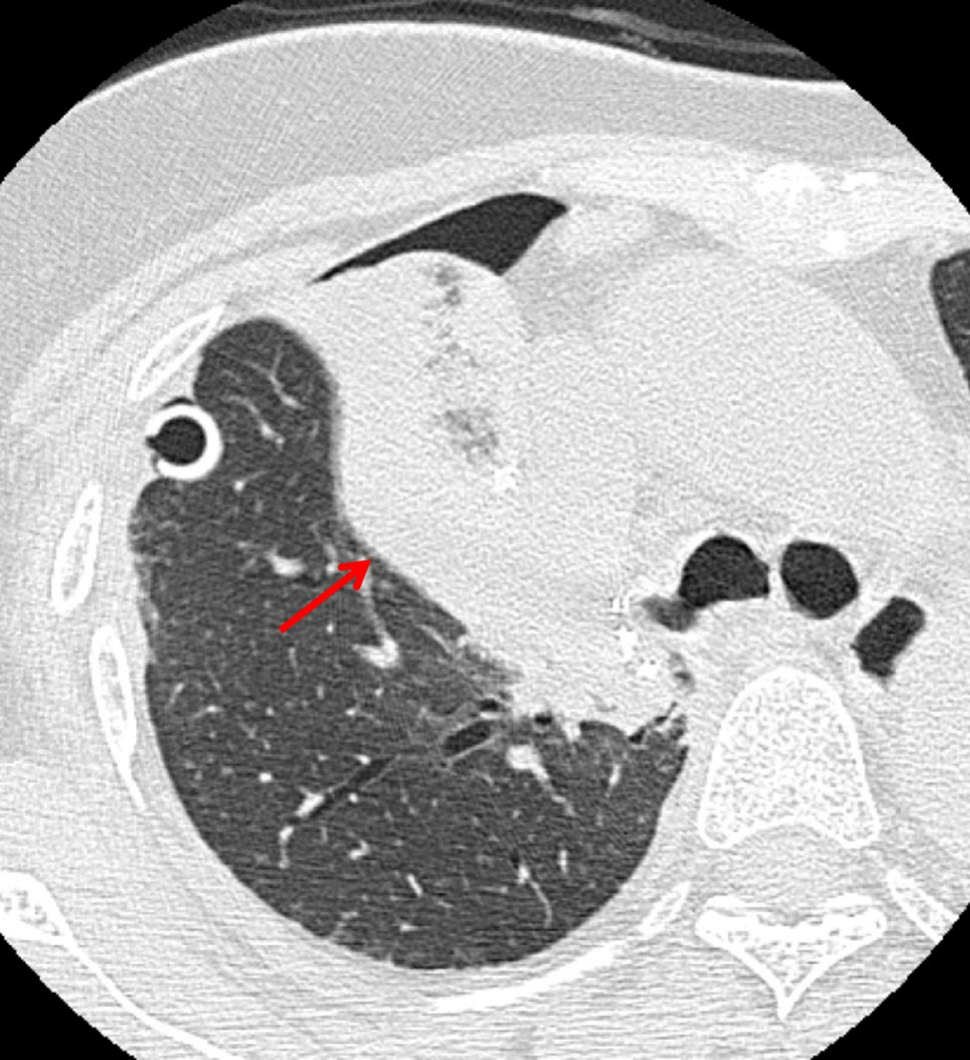
Lung torsion following right upper lobectomy. CT performed 2 days postoperatively shows consolidation in the twisted middle lobe (arrow)

Regarding imaging, CXR should raise suspicion when atelectasis or consolidation appears in an abnormal location. CT demonstrates a twisted lobe with consolidation, GGO, and interlobular/intralobular septal thickening (Fig. 18). Contrast-enhanced CT is essential for detecting vascular tapering, bronchial rotation, and diminished enhancement. Coronal and sagittal reconstructions and 3D imaging further aid diagnosis [8, 35, 36].

Recognizing this life-threatening condition is critical, as it requires emergency surgery to release the torsion or resect the affected lobe.

#### Thrombosis in the pulmonary vein (PV) stump

Thrombosis within the pulmonary vein (PV) stump is a significant concern due to its potential for systemic embolization, including cerebral infarction. This complication may arise at any stage, from days to months postoperatively.

**Fig. 19:**
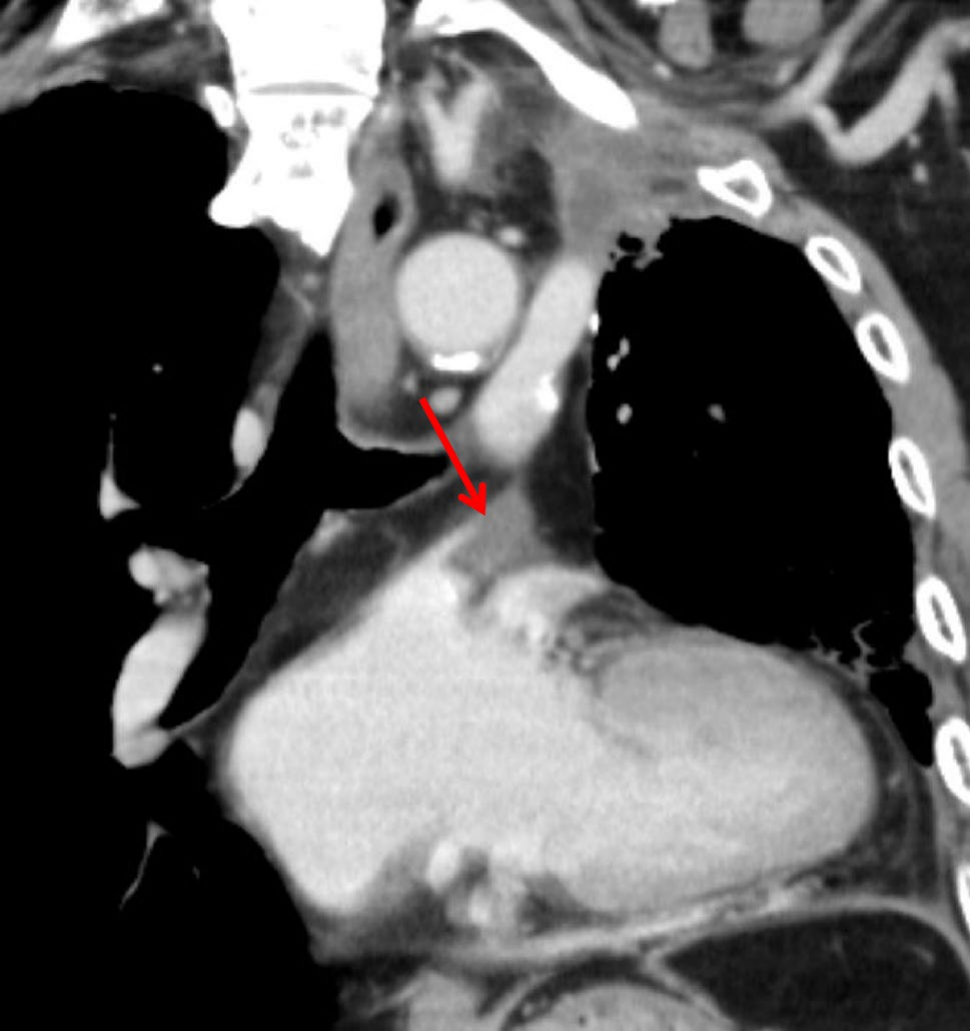
Thrombosis in the pulmonary vein stump. Eight months after left upper lobectomy, contrast-enhanced coronal CT shows a relatively elongated left superior pulmonary vein stump with a filling defect, consistent with thrombus formation (arrow)

A known risk factor is left upper lobectomy, as the left superior PV stump is anatomically longer than the remaining three pulmonary veins, predisposing it to thrombus formation [37–39]. Although a standardized management approach has not been established, anticoagulation may be initiated when a thrombus is identified [40]. Radiologists should routinely evaluate the PV stump on postoperative scans to identify thrombus formation early (Fig. 19).

## Conclusion

This review has outlined the expected postoperative imaging findings and the spectrum of complications that may arise following lung cancer surgery. Postoperative complications vary widely in presentation and severity, ranging from benign self-limiting changes to life-threatening conditions requiring urgent intervention.

Accurate radiologic interpretation is crucial for distinguishing between expected postoperative changes and true pathologic findings. Therefore, a thorough understanding of the radiologic features of normal postoperative changes and complications is essential for avoiding unnecessary diagnostic workups, ensuring early detection, and optimizing patient management.
